# Primary aortoduodenal fistula – overlooked because of guidelines?

**DOI:** 10.1515/iss-2020-0007

**Published:** 2020-10-29

**Authors:** Felix Wiesmueller, Clemens Neufert, Jürgen Siebler, Roland Croner, Werner Lang, Robert Grützmann

**Affiliations:** Department of Surgery, University Hospital Erlangen, Friedrich-Alexander-University of Erlangen-Nuremberg (FAU), Erlangen, Germany; Department of Internal Medicine I, University Hospital Erlangen, Friedrich-Alexander-University of Erlangen-Nuremberg (FAU), Erlangen, Germany; Department of General, Visceral, Vascular and Graft Surgery, University Hospital Magdeburg, Otto-von-Guericke University of Magdeburg, Magdeburg, Germany; Department of Vascular Surgery, University Hospital Erlangen, Friedrich-Alexander-University of Erlangen-Nuremberg (FAU), Erlangen, Germany

**Keywords:** aortoduodenal fistula, aortoenteric fistula, gastrointestinal bleeding, PADF, primary aortoduodenal fistula

## Abstract

Primary aortoduodenal fistula is an uncommon yet mostly lethal finding. We present a case of a 63 year-old male who exhibited significant upper gastrointestinal bleeding and hemorrhagic shock. Repeated endoscopies did not detect any source of bleeding. Emergency laparotomy disclosed an aortoduodenal fistula. Despite intense medical efforts for several months the patient did not fully recover and treatment was limited to palliative care. In light of the substantial mortality associated with this condition, computed tomography imaging should be performed in case of doubt to prevent delayed diagnosis.

## Introduction

Primary aortoduodenal fistula (PADF) is a rare but serious condition in which an abnormal passage between the aorta and the duodenum has been formed. Copious amounts of blood are lost into the duodenum resulting in hemodynamic instability and likely death. The incidence of PADF ranges between 0.04 and 0.07%, although it might be higher than suspected [1], [2]. Upper or lower gastrointestinal (GI) bleeding are common findings in an emergency room setting. Therefore, PADF often is overlooked or the diagnosis is established at a late stage. When untreated, mortality of PADF approaches 100% [3]. Hence, a high index of clinical suspicion must be maintained to diagnose this potentially fatal condition.

## Case report

**Figure 1:**
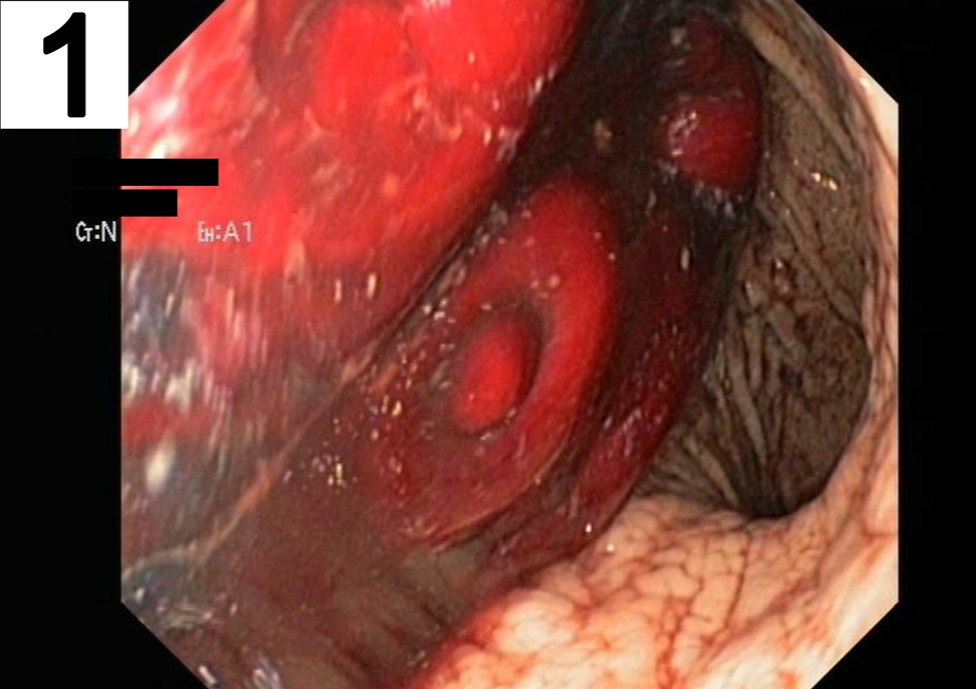
Endoscopic view of the stomach. A large blood clot is seen in the gastric corpus. Yet, no active site of bleeding is visible.

**Figure 2:**
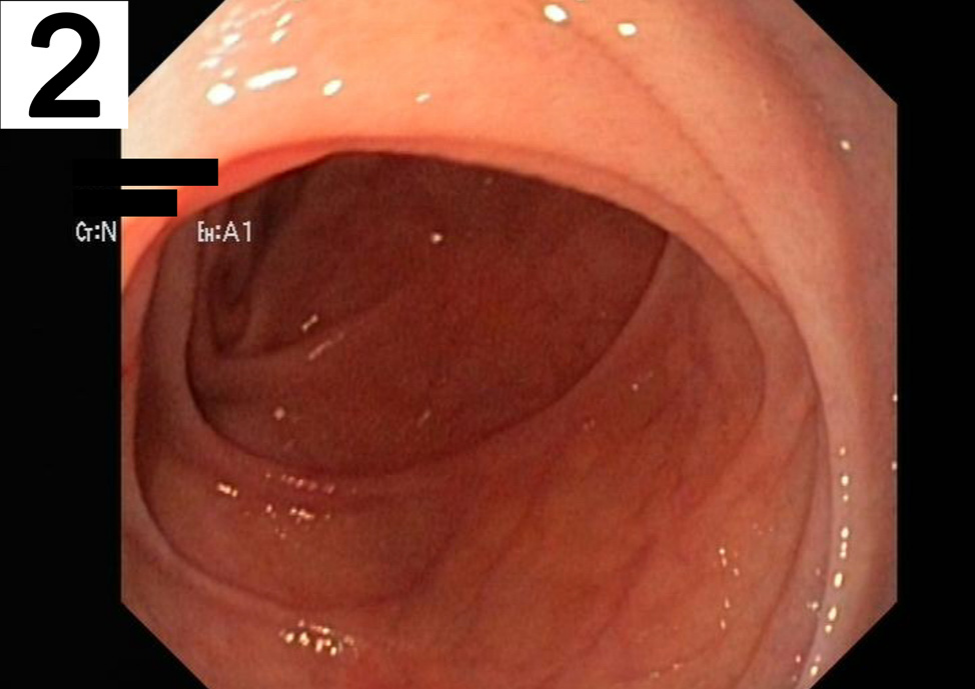
Endoscopic view of the duodenum. No hematoma, blood clot or active bleeding could be identified inside the duodenum.

**Figure 3:**
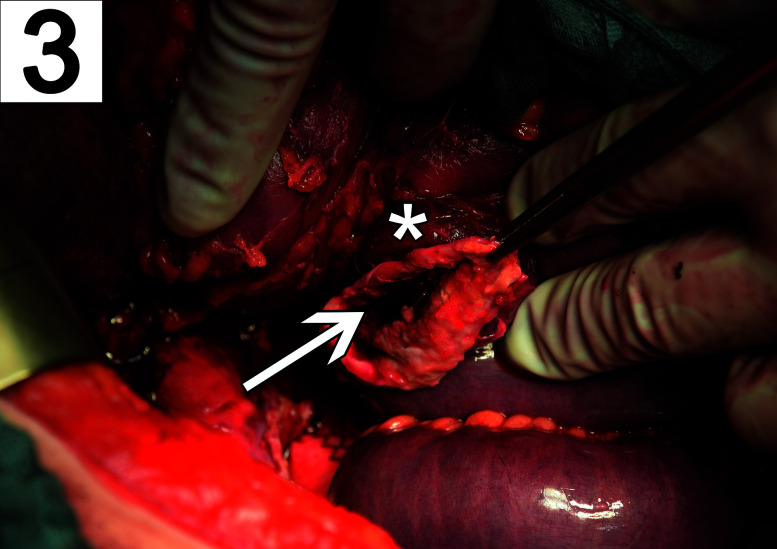
Intraoperative view of the excised fistula (arrow) and the remaining aortic aneurysm sac (grasped by forceps) that is adherent to the posterior duodenum (asterisk).

**Figure 4:**
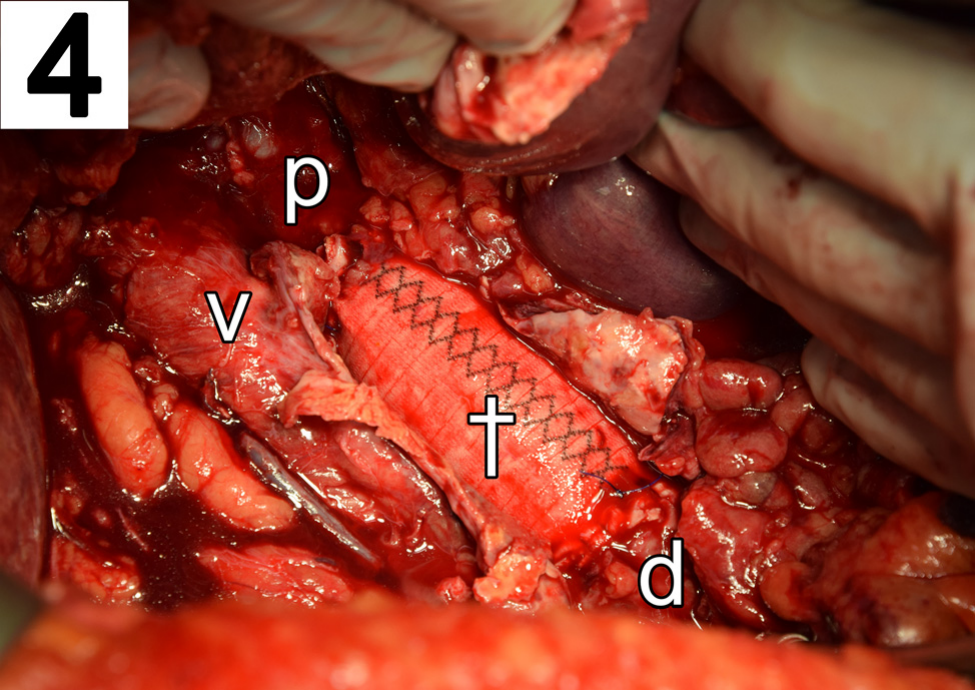
Intraoperative view of the aortic inlay graft (obelisk) residing in the aorta (p: proximal aorta, d: distal aorta) next to the vena cava inferior (v).

A 63 year-old man was transferred to our emergency department for significant gastrointestinal bleeding. Upon arrival, the patient was awake but not oriented. His vital signs included a respiratory rate of 40/min, a pulse of 121 beats/min, a blood pressure of 105/38 mmHg and 100% oxygen saturation at 2 L/min per nasal cannula. Physical examination showed a pale appearing male in severe distress but was otherwise unremarkable. A complete blood count revealed a hemoglobin concentration of 3 g/dL. His medical history included arterial hypertension, alcohol abuse and nicotine dependence. Treatment for hemorrhagic shock was initiated immediately by administering large intravenous volume infusions, transfusions and multiple vasopressors. A focused assessment with sonography for trauma scan was performed. However, the infrarenal part of aorta was not visible due to excessive bowel gas. The patient was intubated and a nasogastric tube was placed which revealed fresh red blood. An emergency esophagogastroduodenoscopy (EGD) was performed, which showed large blood clots in the stomach (Figure 1). However, an active source of bleeding was not present, neither in the stomach nor the duodenum (Figure 2). High-dose proton-pump inhibitors and erythromycin were infused intravenously. Despite rapid fluid replacement, the patient went into acute renal failure and required continuous hemofiltration. The next day, repeat EGD revealed a small gastric erosion which was supplied with a metal clip. Otherwise no source of bleeding could be identified. A few hours later, the patient suffered from repeat hypotension and hematochezia. A colonoscopy demonstrated blood clots in the colon but no source of bleeding. The following days, his condition improved. He had a nonbloody bowel movement and his renal function recovered. For three days, he did not exhibit any signs of bleeding. A scheduled computed tomography (CT) scan was postponed due to improved condition of the patient. However, after five days the patient suddenly developed hematemesis. During emergency EGD, the patient suffered cardiac arrest and required cardiopulmonary resuscitation (CPR). The distal part of the duodenum exhibited several ulcers and a pulsatile bleeding. Intraluminal exsanguination was massive and the exact localization of the bleeding could not be visualized. The patient was transferred to the operating room under continuous CPR for emergency laparotomy. The laparotomy disclosed a distended and partly ischemic small intestine. An abdominal aortic aneurysm of 5.5 cm diameter adherent to the third part of the duodenum was appreciated. The aortoduodenal fistula (Figure 3) was excised and an aortic 24 mm polyester graft was implanted in inlay technique (Figure 4). The patient received intensive care treatment and numerous surgeries for several months. Diffuse bleeding was common and necessitated a total of 25 platelet transfusions, 118 units of fresh frozen plasma, and 173 units of packed red blood cells (PRBCs). Despite these efforts, multiple organ dysfunction evolved. Decision was made to limit treatment to palliative care in accordance with close family members and presumed will of the patient. After 122 days, the patient succumbed to his condition.

## Discussion

PADF is a rare condition. Its characteristics are summarized in Table 1. Most common etiology of this entity is an abdominal aortic aneurysm (AAA) that connects to the duodenum via a fistula. The most common location for aortoduodenal fistula to develop is the third portion of duodenum due to its vicinity to the aorta in the retroperitoneum. PADF is not to be confused with secondary aortoduodenal fistula (SADF) which results from previous aortic aneurysm repair and which is more common than PADF [4]. Symptoms of PADF may resemble those of any upper or lower GI hemorrhage. A classic triad of symptoms (GI bleeding, abdominal pain and a pulsating abdominal mass) has been described. However this triad is present in only 10% of cases [3]. The so-called herald bleed is a key finding that helps in establishing early diagnosis. It is characterized by a phase of subclinical bleeding that is followed by a hemorrhage-free interval ending in sudden exsanguination. The herald bleed is time-limited due to obstruction of the fistula by a temporary thrombus and spasmic contraction of the intestine around the fistula. The interval between an initial minor bleed and massive exsanguination may range from hours to months [1].

**Table 1:** Characteristics of PADF.

Definition and etiology	**Primary aortoduodenal fistula (PADF)** is an abnormal passage between aorta and duodenum. Causes for formation of this passage include abdominal aortic aneurysm (AAA) (most common), tumors, radiation, foreign bodies, or infections. **Secondary aortoduodenal fistula (SADF)** is a passage between aorta and duodenum that, as a complication, results from AAA repair.
Prevalence	0.04–0.07% [2]
Gender	♂:♀ = 3:1 [10]
Symptoms	GI bleeding (hematemesis, melena), abdominal or back pain, abdominal pulsating mass, fever, sepsis, shock, syncope
Diagnostics	Helical CT is test of choice [3]
Treatment	Graft insertion by open or endovascular surgery (no data available on superiority of either approach)
Mortality	Untreated: 100% Perioperative: 30–70% [10]

Diagnostic test of choice is a CT scan with intravenous contrast [3]. However, suspicion for PADF may be initially raised by a bleed that may closely resemble any other cause of upper GI bleeding. Most common cause of an upper GI bleeding is a gastric ulcer, a condition frequently encountered in an emergency department [5]. Therefore, the Practice Guidelines of the American College of Gastroenterology recommend upper endoscopy as the first step in diagnosis and therapy of a suspected ulcer bleed [6]. In overt lower intestinal bleeding, their guidelines advise to first perform lower endoscopy if possible [7]. Both algorithms as well as German guidelines rather prefer endoscopy than CT imaging in early diagnosis [8]. Hence, EGD is chosen most frequently as first diagnostic measure, as this was the case in our patient [3]. Yet, only the proximal portions of the duodenum are frequently visualized [1]. Intestinal folds often conceal the fistula itself. Several authors underline that EGD was not able to identify a source of bleeding in aortoenteric fistula [9], [10]. Moreover, endoscopy may accidentally disrupt a clot and lead to life-threatening exsanguination [10]. On the other hand, CT scans expose patients to ionizing radiation and potentially pose a health risk if used on a regular basis. Given the low prevalence of PADF, more patients would be harmed by radiation than safe by routine CT scans.

Correct diagnosis could have been made earlier in the presented case. In hindsight, guideline algorithms and the hemorrhage-free interval after the herald bleed may have led to not insisting on immediate CT imaging. Although algorithms do not exclude the usage of clinical judgment, they might distract from doing so. Above all, successful diagnosis of PADF results from a very high level of clinical suspicion. With this case report we want to remind the reader of (1) the difficulty in not confusing PADF with an ulcer or variceal bleed and of (2) the hemorrhage-free interval following the sentinel bleed which might lead to inertia in pushing further diagnostics.

Taking everything into account, we advocate considering CT imaging early in case of doubt in patients with obscure and massive upper GI bleeding, even if CT imaging is not highlighted by current guideline algorithms.
