# Supplementary material for: Primary aortoduodenal fistula – overlooked because of guidelines?
Source: Innov Surg Sci. 2020 Oct 29;5(3-4):133–6. doi: 10.1515/iss-2020-0007 (PMC8668031; doi:10.1515/iss-2020-0007)
Supplement: Supplementary file 1 [file iss-05-20200007-s001.pdf]

Reviewer Assessment

Felix Wiesmüller et al.: Primary Aortoduodenal Fistula – overlooked because of guidelines?

Reviewers' Comments to Original Submission

Reviewer 1: anonymous

Date received: 13-May-2020
Reviewer recommendation: **Return to author for major modifications**
Reviewer overall scoring: **Low**

Assessment Form scores: 5 = High/Yes; 3 = Medium/Adequate; 1 = Low

Is the subject area appropriate for the journal					
Does the title clearly reflect the paper's content?					1
Does the abstract clearly reflect the paper's content			3		
Do the keywords clearly reflect the paper's content?			3		
Does the introduction present the problem clearly?					1
Are the results/ conclusions justified?	5				
How comprehensive and up-to-date is the subject matter presented?				2	
How adequate is the data presentation?				2	
Are units and terminology used correctly?			3		
Is the number of cases adequate?					1
Are the experimental methods/ clinical studies adequate?			3		
Is the length appropriate in relation to the content?			3		
Does the reader get new insights from the article?				2	
Please rate the practical significance.					1
Please rate the accuracy of methods.			3		
Please rate the statistical evaluation and quality control.			3		
Please rate the appropriateness of the figures and tables.			3		
Please rate the appropriateness of the references.					1
Please evaluate the writing style and use of language.			3		
Please judge the overall scientific quality of the manuscript.				2	
Are the methods used worthy of reproduction in greater deal?			No		
Would you be willing to review a revision of this manuscript?			No		

Comments to author: This paper should only be published as an indication of how not to do it.The most common cause of a primary aortoduodenal fistula is an abdominal aortic aneurysm - and this was also the case here. A CT scan should have been performed at the latest on the day after admission, this does not prohibit any guideline. The paper must clearly point out the deficiencies in diagnostics.

Besides:-
- The abstract and paper are not structured.
- The literature search is superficial
- A detailed discussion of the literature is missing.

Reviewer 2: Busch, Thomas

Date received: 07-Jul-2020
Reviewer recommendation: **Accept in present form**
Reviewer overall scoring: **Medium**

Assessment Form scores: 5 = High/Yes; 3 = Medium/Adequate; 1 = Low

Is the subject area appropriate for the journal		4			
Does the title clearly reflect the paper's content?		4			
Does the abstract clearly reflect the paper's content		4			
Do the keywords clearly reflect the paper's content?		4			
Does the introduction present the problem clearly?		4			
Are the results/ conclusions justified?		4			
How comprehensive and up-to-date is the subject matter presented?			3		
How adequate is the data presentation?		4			
Are units and terminology used correctly?		4			
Is the number of cases adequate?		4			
Are the experimental methods/ clinical studies adequate?		4			
Is the length appropriate in relation to the content?		4			
Does the reader get new insights from the article?				2	
Please rate the practical significance.				2	
Please rate the accuracy of methods.		4			
Please rate the statistical evaluation and quality control.		4			
Please rate the appropriateness of the figures and tables.		4			
Please rate the appropriateness of the references.		4			
Please evaluate the writing style and use of language.		4			
Please judge the overall scientific quality of the manuscript.				2	
Are the methods used worthy of reproduction in greater deal?	No response				
Would you be willing to review a revision of this manuscript?	Yes				

Comments to author: With admission to hospital emergency esophagogastroduodenoscopy was performed as well as abdominal sonography. No cause of bleeding could be detected and the infrarenal part of the aorta could not be assessed. At that time a CT-scan should have been enforced. Due to patients medical history and due to the clinical situation you described an abdominal CT-scan could have possibly been performed. The abdominal aneurysm you detected during emergency laparotomy could have been diagnosed at the day of admission. The differential diagnosis of a possible aortoduodenal fistula could be established much more earlier. A five-day- interval from admission to diagnosis during laparotomy was to long in the described situation.

Authors' Response to Reviewer Comments

Date received: DD-Mon-2020

Response to reviewer 1

Dear reviewer, we appreciate your constructive revision. We modified the discussion to include the fact that the most common cause of PADF is an abdominal aortic aneurysm (highlighted in yellow). Also, we modified the last part of the discussion to include the deficiencies that led to delayed diagnosis in our patient case (highlighted in blue since reviewer 2 commented on this as well).

Regarding your bullet points:

(1): In accordance to the "Information for Authors" by the publisher, the abstract remains unstructured. We structured the main part into Introduction, Case Report and Discussion.

(2+3): Literature search and discussion of literature remain somewhat superficial since the "Information for Authors" limits the number of references to equal or less than ten. In order to account for your remark, we further discussed literature as the number of references permitted (highlighted in yellow).

Response to reviewer 2

Dear reviewer, thank you for your helpful comments. You are right: a CT scan could have been performed from early on. Instead, proper diagnosis was delayed. The patient did not receive treatment at the time that would have prevented this unfortunate clinical course and, ultimately, saved his life. The difficulty in this case was that the risk factor profile (smoking, EtOH) was very suggestive of an ulcer or variceal bleed. The fact that the endoscopist placed a clip and the patient became rather stable over the initial course of his hospital stay (aka the hemorrhage-free interval) must have led to not insisting on immediate CT imaging. In accordance to your comments, we modified the discussion to underline the pitfalls in diagnosing this entity (highlighted in blue).

Comments by the Editor-in-Chief to Revised Submission

All reviewer concerns raised were addressed satisfactorily. The manuscript may be published in the present stage.
